# Effects of Low-Light Environments on the Growth and Physiological and Biochemical Parameters of *Indocalamus* and Seasonal Variations in Leaf Active Substance Contents

**DOI:** 10.3390/plants12233993

**Published:** 2023-11-27

**Authors:** Weiqian Yu, Mingyan Jiang, Qiling Yue, Yixiong Yang, Zhenghua Luo, Bingyang Lv, Rui He, Shihan Feng, Meng Yang

**Affiliations:** College of Landscape Architecture, Sichuan Agricultural University, Chengdu 611130, China

**Keywords:** *Indocalamus*, low-light environment, growth adaptation, understory plants, active substances

## Abstract

*Indocalamus*, characterized by its expansive leaves, low height, strong reproductive capacity, and abundant bioactive compounds, has extensive utility in the realms of food processing, the manufacturing of packaging materials, and the advancement of novel pharmaceuticals. Two light environments, CK (100% full light) and ST (50% full light), were established to explore the effects of low-light environments on the reproductive ability, morphological characteristics, photosynthetic properties, and leaf active substances of 14 *Indocalamus* species. The findings revealed that in comparison to the CK treatment, for 14 species of *Indocalamus* under the ST treatment, (1) the diameter, single leaf area, and leaf area index increased by 8.27%, 8.14%, and 17.88%, respectively; (2) the net photosynthetic rate decreased by 15.14%, and the total chlorophyll contents increased by 20.25%; and (3) the total flavonoid contents increased by 18.28% in autumn, the total polyphenol contents increased by 48.96% in spring, and the total polysaccharide contents increased by 31.44% and 30.81% in summer and winter, respectively. In summary, *Indocalamus* are adapted to survive in low-light environments; the growth and physiological indices differ significantly between the two light environments, and the low-light environment can effectively promote the growth and development of the leaves. Furthermore, the leaves are rich in flavonoids, polyphenols, polysaccharides, and active substances, which are affected by the light intensity and the season to varying degrees, and autumn and winter are the best times for harvesting the leaves. The leaves of *I. hunanensis* and *I. lacunosus* are richest in flavonoids and polyphenols, while the leaves of *I. kunmingensis* cv. fuminer are richest in polysaccharides. The main findings of this study demonstrate that *Indocalamus* has strong shade tolerance and tremendous leaf value, laying the foundation for broadening the application of their leaves and for their industrial development in understory composite planting systems.

## 1. Introduction

*Indocalamus* belongs to the Bambusoideae (Gramineae), which is a class of dwarf bamboo with large evergreen leaves and landscaping value [1]. They have rapid growth rates, high yields, and high reproductive capacity, and are easy to manage after planting [2]. *Indocalamus* has a wide range of uses; their culms can be used to make bamboo chopsticks, pen culms, and broom handles, among other things, and they are regarded as one of the most significant resources for the development of flavonoid products due to the high flavonoid contents in their leaves and the ease with which the flavonoids can be extracted, isolated, and purified [3,4]. Moreover, the leaves of *Indocalamus* are a rich source of bioactive compounds such as polyphenols, polysaccharides, chlorophylls, and various aromatic components [5,6] with anticancer, antitumor, and antioxidant effects as well as antibacterial properties [7,8]. In recent years, *Indocalamus* leaves have been extensively utilized in food processing, packaging material manufacturing, and the development of new drugs [9,10]. *Indocalamus* is not only a group of bamboo species with excellent leaf uses but also ideal greening plants for understory planting, with broad application value and development potential.

Understory planting enhances land utilization by maximizing vertical space, which not only reduces planting costs but also improves soil biodiversity and ecosystem function [11]. Whereas the light transmission rate in gaps within the forest is an important factor affecting the survival, growth, regeneration, and ultimate productivity of understory plants, light is an environmental signal that regulates the growth, development, and morphogenesis of plants [12]. Common groundcover bamboo species, *Indocalamus*, are adaptive to changes in light [13], producing a series of regulatory mechanisms to improve light capture efficiency in low light, allowing plants to adapt to low light quantum densities and maintain normal biological functions [14,15,16]. The leaves of the *Indocalamus* are large, and the leaves are the main organs for various physiological and metabolic activities. Due to phenotypic plasticity, the leaves usually show significant morphological and physiological regulation under different light environments [17], and the regulation of light intensity within an appropriate range can aid *Indocalamus* leaf growth [18].

The production of bioactive compounds in plants is believed to be influenced by numerous factors, with individual plant development being the most significant [19]. Light intensity can affect individual plant development by influencing life activities such as photosynthesis and respiration, and modulation of light conditions can influence the synthesis and catabolic processes of plant active substances [20]. The accumulation of bioactive compounds in plants is closely related to seasonal changes, which alter the climatic factors of the plant growth environment, thereby affecting the growth and physiological and metabolic activities of plants, and plants alter their types and contents of bioactive compounds in their structures in response to climate change [21]. Light intensity and seasonal changes can synergistically inhibit or stimulate the ability of *Indocalamus* to synthesize bioactive compounds, affecting the production of leaf active substances and the time at which the leaf active substance contents reach their annual peak, thereby delaying or advancing the optimal harvesting period for leaves [22].

This study was closely integrated with the potential application environment of *Indocalamus* and was carried out to investigate the growth physiology and leaf active substances of 14 species of *Indocalamus* in two categories of light environments. The aim was to reveal the growth, physiological, and biochemical responses of these species to the low-light environment and the dynamic change patterns in leaf active substances and to determine the optimal harvest time of the leaves.

## 2. Materials and Methods

### 2.1. Plant Materials and Growth Conditions

**Figure 1:**
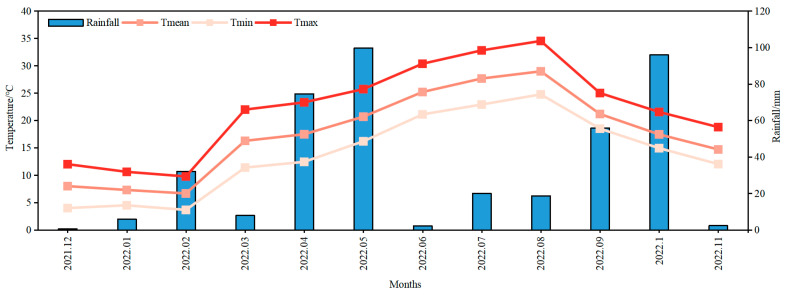
Mean temperature and precipitation during the experimental period.

The 14 *Indocalamus* species used in the experiment were all from Asia (Table A1). For all species, 2 year old split seedlings were used. The experimental site is located in the southwestern region of China (30°42′35.58″ N, 103°47′49.06″ E), which has a humid subtropical monsoon climate characterized by four distinct and pleasant seasons, with high temperatures and rainfall during the summer, while the winter season is mild and humid (Figure 1). The nursery consisted of a raised bed comprising a total of 28 planting plots, with each species of *Indocalamus* occupying two plots. Each planting plot measured 3 m in length and 1.2 m in width, resulting in a total area of 3.6 m^2^. Additionally, an isolation ditch with a width of 50 cm and a depth of 20 cm was established between each planting plot. Each plot was planted with 3 clusters of identical species of *Indocalamus*, and the number of standing bamboos (i.e., maternal bamboo) per cluster was 10–12. The maternal bamboos of different species exhibited variations in body volumes, consequently resulting in varying quantities of maternal bamboos during the planting process. A total of 28 planting plots were divided into two groups, with 14 plots receiving the 100% full light treatment and the remaining 14 plots receiving the 50% full light treatment. Shade nets were used to shade the top and periphery of the 14 planting plots in the shade treatment, and the shade effect in the 50% full light treatment was achieved by adjusting the light transmission slits of the nets. The soil in the nursery is composed of yellow loam with a pH of 6.82, containing 20.3 mg·kg^−1^ of organic matter, 238.0 mg·kg^−1^ of effective nitrogen, 116.3 mg·kg^−1^ of fast-acting phosphorus, and 76.1 mg·kg^−1^ of fast-acting potassium.

### 2.2. Experimental Design

From December 2021 to November 2022, two light environments, CK (100% full light) and ST (50% full light), were set up for 14 species of *Indocalamus*. In October 2022, the number of newly grown bamboos of *Indocalamus* was counted after a single growth cycle, and the heights, diameters, area of a single leaf, leaf area index (LAI), net photosynthetic rate (P_n_), total chlorophyll contents (TChl), and chlorophyll a/b ratio (Chl a/b ratio) were determined. The investigation conducted during the dynamic observation phase involved the determination of the total flavonoid (TFC), total polyphenol (TPC), and total polysaccharide (TPS) contents of each bamboo species in winter (January 2022), spring (April 2022), summer (July 2022), and autumn (October 2022).

### 2.3. Measurement Methods

#### 2.3.1. Observation of the Number of Bamboo Shoots

The young bamboos aged 2 to 3 years with good growth were the maternal bamboos; new bamboo shoots were grown during the shooting period (February to May), and the number of new bamboos was counted after the new shoots had become bamboos. New bamboo rate = (the number of new bamboos per clump ÷ the number of maternal bamboos per clump) × 100%.

#### 2.3.2. Leaf Morphology and Quantification

Fully grown bamboo with fully extended leaves and uniform growth in four directions was randomly selected per treatment. A total of 12 new bamboos were selected for this study. Specifically, the median leaves from the middle and upper twigs that were healthy and undamaged were chosen. The outlines of these leaves were then meticulously drawn, and their areas were measured using AutoCAD software (Ver. 2019, Autodesk Corp., San Francisco, CA, USA). The LAI was quantified using the LAI-2200 canopy analyzer (America, LI-COR Corp., Lincoln, NE, USA).

#### 2.3.3. Photosynthetic Parameters

The P_n_ (μmol CO_2_·m^−2^·s^−1^) was determined for 28 treatments and 14 species of *Indocalamus* using the LI-6400XT portable photosynthesis measurement system (America, LI-COR Corp.). The TChl contents and Chl a/b of leaves were calculated using the equations of Lichtenthaler and Wellburn [23], and the tested leaves were selected in the same manner as in Section 2.3.2.

#### 2.3.4. Leaf Active Substances

The rutin colorimetric method described by Chen [24], the Folin–Ciocalteu method described by Ainsworth [25], and the phenol-concentrated sulfuric acid method described by Gao [26] were used, and the TFC, TPC, and TPS contents were calculated for each treatment based on the corresponding standard curves. TFC and TPC contents were determined using dry leaves, while TPS contents were determined using fresh leaves. The tested leaves were selected in the same manner as described in Section 2.3.2.

### 2.4. Statistical Analysis

Data were processed by SPSS (Ver. 26.0, IBM Corp.). One-way ANOVA and Duncan’s multiple comparison analysis test (*p* ≤ 0.05) were used to test for significant differences; Pearson correlation analysis was used to test for associations between variables; and Excel (Ver. 2019, Microsoft Corp., Redmond, WA, USA) and Origin (Ver. 2023b, OriginLab, Northampton, MA, USA) were used to construct graphs.

## 3. Results

### 3.1. Growth Characteristics and Photosynthetic Capacity of Indocalamus

**Figure 2:**
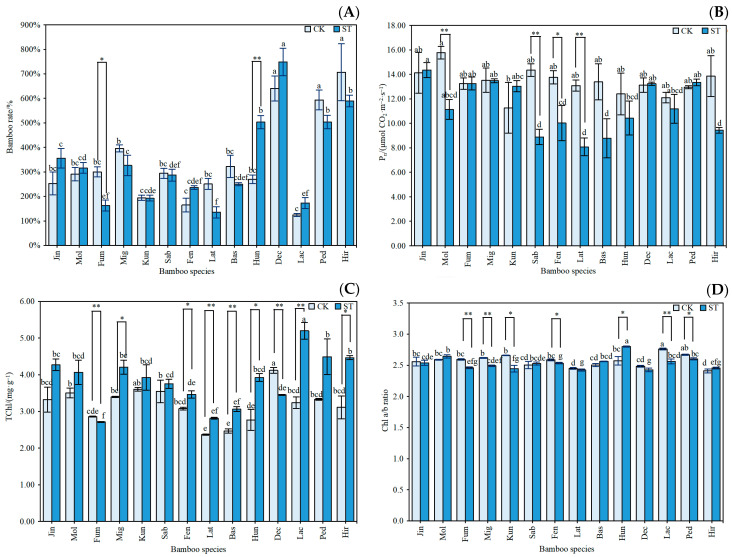
Responses of (**A**) the new bamboo rate, (**B**) Pn, (**C**) TChl, and (**D**) Chl a/b ratio to different light environments in *Indocalamus.* The data in the graphs are the means ± standard errors (SE); based on Duncan’s multiple comparisons method, different lowercase letters indicate significant differences among different bamboo species under the same light environment (*p* < 0.05); ** indicates highly significant differences among the same bamboo species under different light environments (*p* < 0.01), and * indicates significant differences among the same bamboo species under different light environments (*p* < 0.05).

Figure 2 depicts the effects of the two light environments on the rate of new bamboo and the photosynthetic capacity of *Indocalamus*. Compared with those in the CK treatment, the new bamboo rates of the 14 species of *Indocalamus* under the ST treatment differed by −46.03% to 43.34%, with a large difference between bamboo species. Under the ST treatment, the average P_n_ of the 14 species of *Indocalamus* decreased by 15.14% compared with that of the CK treatment, and the P_n_ of only four species of *Indocalamus* increased. Except for Fum and Dec, the TChl contents were higher in the bamboo under the ST treatment than under the CK treatment. The Chl a/b ratio of *Indocalamus* was less than three in both light environments, and the ST treatment reduced the Chl a/b ratio by 1.35% compared with that in the CK treatment, and all bamboo species were able to use dispersed light effectively.

### 3.2. Morphological Characteristics of Indocalamus

**Figure 3:**
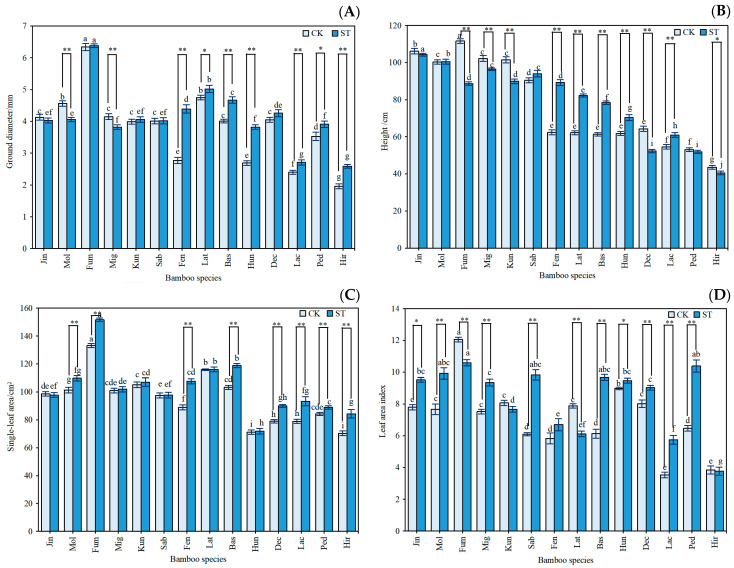
Response of (**A**) ground diameter, (**B**) height, (**C**) single leaf area, and (**D**) LAI to different light environments in *Indocalamus.* The data in the graphs are the means ± standard errors (SE); based on Duncan’s multiple comparisons method, different lowercase letters indicate significant differences among different bamboo species under the same light environment (*p* < 0.05); ** indicates highly significant differences among the same bamboo species under different light environments (*p* < 0.01), and * indicates significant differences among the same bamboo species under different light environments (*p* < 0.05).

Figure 3 depicts the effects of the two light environments on the morphological characteristics of *Indocalamus*. Under the ST treatment, the average ground diameter and the average height of the 14 species of *Indocalamus* increased by 8.27% and 2.26%, respectively, compared with the CK treatment. Except for Mol, Mig, and Jin, the ground diameter of the bamboo species was larger under the ST treatment than under the CK treatment. The coefficient of variation was moderate at 10% to 20%, higher at 20% to 30%, and very high at greater than 30% [27]. Under the CK treatment, the coefficients of variation in the ground diameter (29.47%) and height (30.38%) of *Indocalamus* were larger, showing higher abundance, which indicated that the thickness and height of the bamboo poles varied greatly, whereas the coefficients of variation in the ground diameter (22.92%) and height (25.40%) of *Indocalamus* in the ST treatment were reduced, indicating that the low-light environment reduced the degree of morphometric variation.

For all 14 species of *Indocalamus*, the single leaf areas were larger under the ST treatment than under the CK treatment; the coefficient of variation was moderate (19.44%), and the degree of difference in the single leaf area was lower than that for the height and diameter, indicating that it was more stable. Under the ST treatment, the single leaf areas of 10 species were larger than 100.00 cm^2^, indicating that these species are suitable for food packaging material manufacturing. Except for Lat, Fum, and Kun, the LAI of the bamboo species increased by 5.46% to 62.89% under the ST treatment compared with the CK treatment, and the LAIs of 12 species of *Indocalamus* under the ST treatment were larger than six, with significant numbers of leaves.

### 3.3. Dynamics of Leaf Active Substances

**Figure 4:**
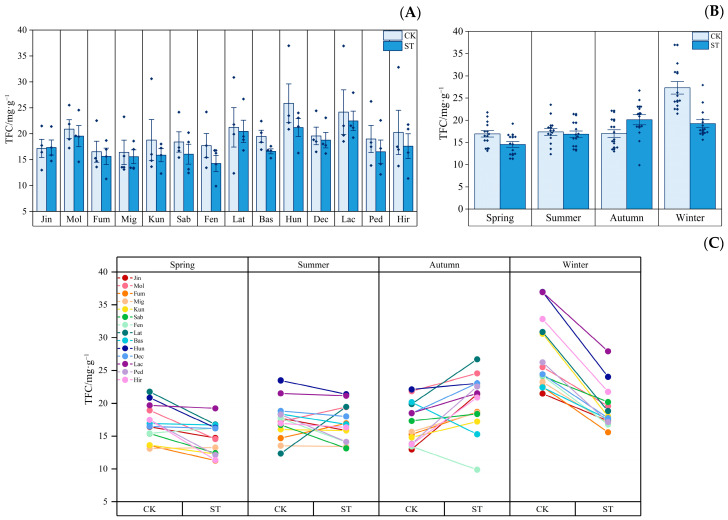
(**A**) Average annual TFC contents of each *Indocalamus* species in the CK and ST treatments; (**B**) average TFC contents in each season in the CK and ST treatments; (**C**) before and after change in the TFC contents in *Indocalamus* in the CK and ST treatments. The diamond dots in (**A**) indicate the mean values of each of the 14 species of *Indocalamus* in the four seasons.

Figure 4 depicts the seasonal dynamics of the TFC contents of *Indocalamus* in two light environments. Figure 4A shows that under the ST treatment, the average annual TFC contents of all the *Indocalamus* except Jin were reduced by 3.68% to 19.45% compared with those under the CK treatment. Figure 4B shows that the average TFC contents of the 14 species of *Indocalamus* in the CK treatment reached their peak in winter (27.31 mg·g^−1^), the ST treatment promoted the accumulation of average TFC (20.13 mg·g^−1^) in the 14 species of *Indocalamus* in autumn, which was 18.28% higher than that in the CK treatment, and suppressed the accumulation of TFC in the other three seasons, and the accumulation of average TFC in winter was the most affected and decreased by 29.53% compared with that in the CK treatment. Figure 4C shows that under the ST treatment, the TFC contents of 12 species of *Indocalamus*, excluding Fen and Bas, in autumn increased by 4.14% to 64.78% compared with the CK treatment.

**Figure 5:**
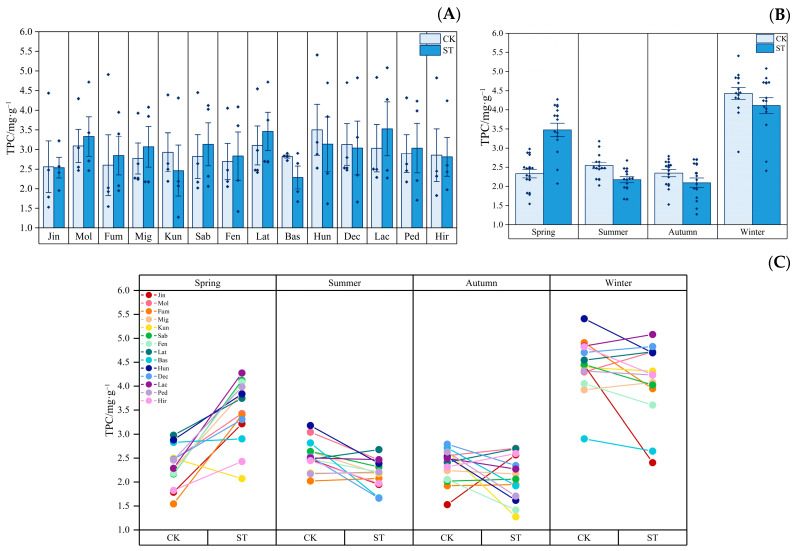
(**A**) Average annual TPC contents of each *Indocalamus* species in the CK and ST treatments; (**B**) average TPC contents in each season in the CK and ST treatments; (**C**) before and after change in the TPC contents of *Indocalamus* in the CK and ST treatments. The diamond dots in (**A**) indicate the mean values of each of the 14 species of *Indocalamus* in the four seasons.

Figure 5 depicts the seasonal dynamics of the TPC contents of *Indocalamus* in two light environments. Figure 5A shows that, compared with the CK treatment, the annual average TPC contents of eight species of *Indocalamus* increased by 4.75% to 16.29% under the ST treatment. Figure 5B shows that under the CK and ST treatments, the average TPC contents of 14 species of *Indocalamus* peaked in winter, with values of 4.429 mg·g^−1^ and 4.110 mg·g^−1^, respectively. Under the ST treatment, the average TPC contents of 14 species of *Indocalamus* increased by 48.96% in spring compared with that in the CK treatment, while it decreased significantly in the other three seasons; in particular, the TPC contents accumulation in summer was inhibited significantly and was 14.56% lower than that in the CK treatment. Figure 5C shows that, compared with the CK treatment, the TFC contents of 13 species of *Indocalamus* (excluding Kun) in spring increased by 2.57% to 119.96% under the ST treatment.

**Figure 6:**
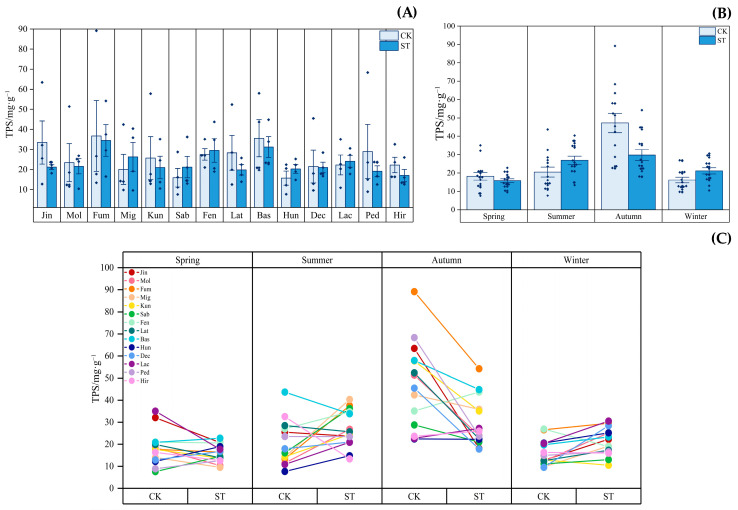
(**A**) Average annual TPS contents of each *Indocalamus* species in the CK and ST treatments; (**B**) average TPS contents in each season in the CK and ST treatments; (**C**) before and after change in the TPS contents in *Indocalamus* in the CK and ST treatments. The diamond dots in (**A**) indicate the mean values of each of the 14 species of *Indocalamus* in the four seasons.

Figure 6 depicts the seasonal dynamics of the TPS contents in *Indocalamus* in two light environments. Figure 6A shows that the annual average TPS contents of nine species of *Indocalamus* were reduced by 7.91% to 45.96% under the ST treatment compared with those under the CK treatment. Figure 6B shows the average TPS contents of 14 species of *Indocalamus* peaked in autumn under both the CK and ST treatments, with high values of 47.206 mg·g^−1^ and 29.736 mg·g^−1^, respectively. The average TPS contents of 14 species of *Indocalamus* increased by 31.44% and 30.81% in summer and winter, respectively, under the ST treatment, and in autumn, those were significantly reduced by 37.02% compared with those in the CK treatment. Figure 6C shows that, compared with the CK treatment, under the ST treatment, the TPS contents of 11 species of *Indocalamus* decreased by 0.63% to 71.45% in autumn and the TPS contents of 10 species of *Indocalamus* increased by 11.16% to 198.05% in winter.

### 3.4. Correlation Analysis among Indicators

**Figure 7:**
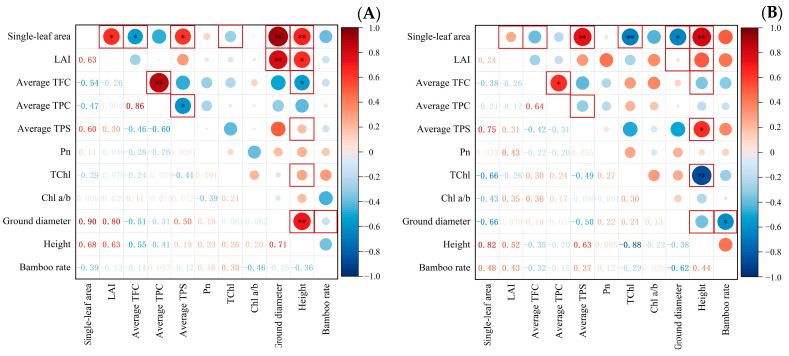
Correlation analysis between indicators of *Indocalamus* under the (**A**) CK treatment and (**B**) ST treatment. The numbers show the correlation coefficients and the color intensity is proportional to the correlation coefficients: blue indicates a positive correlation, red indicates a negative correlation, and * and ** indicate significant differences at *p* < 0.05 and *p* < 0.01, respectively.

Figure 7 depicts the results of the correlation analysis among the indicators of *Indocalamus*. After ST treatment, the single leaf area was no longer correlated with LAI and TPC contents and had a significant negative correlation with TChl contents (r = −0.66); the highly significant positive correlation with ground diameter (r = 0.90) changed to a significant negative correlation (r = −0.66); and LAI was no longer correlated with ground diameter or height. Furthermore, the correlation between the TFC and TPC contents (r = 0.64) was weakened, while TPC and TPS contents were no longer correlated, and TPS contents and height (r = 0.64) were no longer correlated. The correlation between TFC and TPC contents (r = 0.64) was weakened, while TPC and TPS contents were no longer correlated and TPS contents and height (r = 0.63) were significantly positively correlated; TChl contents and height (r = −0.88) were significantly negatively correlated, and ground diameter and height were no longer correlated but were significantly negatively correlated with the new bamboo rate (r = −0.62).

### 3.5. Principal Component Analysis of the Active Substances and Cluster Analysis of the Indicators

**Figure 8:**
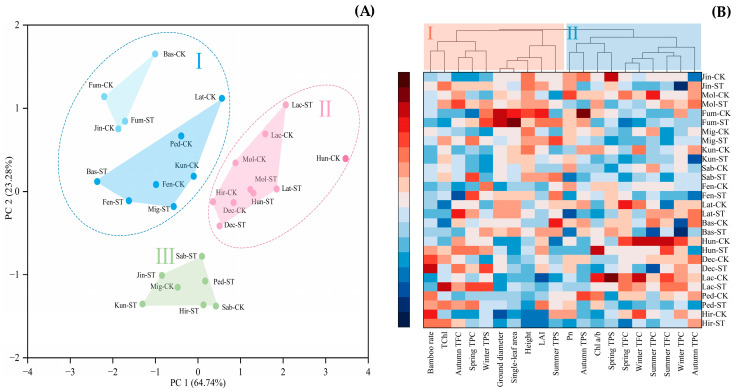
(**A**) Principal component analysis plot and (**B**) clustering heatmap for each indicator of the contents of active compounds in *Indocalamus* in the CK treatment and ST treatment. Indicator data were standardized using the linear normalization method, with each column representing a trait and each row representing a species. Darker red indicates higher values and darker blue indicates lower values.

Figure 8 depicts the principal component analysis of the active substances of *Indocalamus* and the cluster analysis of the samples and traits under the two light environments. Figure 8A shows that, by principal component analysis, *Indocalamus* was classified into three clusters, of which clusters I and II contained two subgroups. Eleven samples were classified as class II, with a higher abundance of TPS and average levels of the other two active ingredients; ten samples were classified as class II, which was characterized by higher abundances of TFC and TPC and a lower abundance of TPS; and seven samples were classified as class III, with no obvious advantage among the active substances. The CK-treated and ST-treated samples of six species of *Indocalamus* (Jin, Mig, Kun, Lat, Ped, and Hir) were located in two different clusters, suggesting that the active substances in vivo fluctuated under different light environments, whereas the remaining species of *Indocalamus* (Mol, Fum, Sab, Fen, Bas, Hun, Dec, and Lac) were close to each other and located in the same clusters, indicating that the ST treatment had less effect on the contents and compositions of their active substances.

The clustered heatmap analysis of *Indocalamus* revealed the differences in growth and physiological indices in the two light environments. Based on Figure 8B, class I comprises 10 indices that experienced a certain degree of increase under the ST treatment, and class II comprises 10 indices that experienced a certain degree of decrease under the ST treatment. Under both treatments, Hun and Lac had a significant advantage in the contents of active substances, and Fum had a significant advantage in terms of reproductive capacity and morphological indicators.

## 4. Discussion

### 4.1. Growth Morphology and Photosynthetic Capacity of Indocalamus in Response to Different Light Environments

Light is not only a necessary prerequisite for plants to photosynthesize but also has a regulatory effect on plant growth, affecting the production of certain hormones in plant structures as well as the morphology, physiology, metabolism, and gene regulation of plant growth [28,29]. Indocalamus plants have low growth rates, and their growth is affected by environmental influences, particularly light intensity [30]. The period in which new bamboo is generated is an essential stage in the bamboo growth cycle, and the rate of new bamboo generation is one of the most significant indicators for assessing the reproductive ability of bamboo plants [31]. In the present study, the reduction in the new bamboo rate of some Indocalamus species under the ST treatment compared with that under the CK treatment may be due to the effect of the low light availability on the photosynthetic carbon sequestration capacity of leaves at the late stage of new shoot growth, leading to insufficient nutrient resources for the generation of new bamboo [32].

Shade-tolerant plants will allocate more of their limited energy to the aboveground parts to obtain more light quanta, thus enabling them to adapt to low-light environments. Usually, plant height and leaf area increase under low light conditions to allow plants to obtain more light energy and decrease light energy absorption under high light conditions to reduce or prevent photoinhibition. In this study, all the *Indocalamus* in the low-light environment showed an increase in leaf area; the heights and diameters of the bamboo generally increased, but there were some differences in the trends of the morphological indices of *Indocalamus*. These results are similar to those of March [33], who analyzed the effects of a low-light environment on the bamboo species of three bamboo subfamilies, and Mulkey [34] and Yang [35], who also found that the low-light environment could increase the leaf area and the number of understory dwarf bamboos of the species *Streptochaeta sodiroana* and *Sinarundinaria nitida*.

Leaves growing in the understory adapted to the reduced light intensity by increasing their light absorption capacity, and the low-light environment in this study could significantly promote the synthesis of TChl in *Indocalamus*, which increased the green landscaping value to some extent. In contrast, P_n_ in the low-light environment was lower than that in the full light treatment, similar to the findings of Terashima [36] and Fan [37]. Since TChl in bamboo plants is in a constant dynamic of synthesis and catabolism, the concentration at which synthesis and catabolism attain equilibrium is lower in high light than in low light, leading to an increase in TChl contents with decreasing light intensity. Lei [38] found that an understory-grown dwarf bamboo, *Sasa senanensis*, exhibited a lower Chl a/b ratio during the summer season, and Smith [39] discovered that the leaf Chl a/b ratio of plants adapted to low-light conditions was lower than 3:1. These results are similar to the findings of the current study. The low-light conditions likely caused the PSI-light-harvesting complex I (LHCI) supercomplex of the shade plants to contain more LHC proteins and the maximal quantum efficiency of PSII to increase, resulting in a low level of Chl a/b ratio in the leaf [40].

### 4.2. Response of Active Substances in the Leaves of Indocalamus to Different Light Environments

There were several significant correlations between growth indices and leaf active compounds (TFC, TPC, and TPS) of *Indocalamus* in this study, which suggests that biological compounds related to the growth of *Indocalamus* may be involved in the same biological activities and vary as a result of light exposure. Su [41] has demonstrated that the leaf total flavonoid contents of the three species of *Indocalamus* ranged between 1.7% and 2.7% in different seasons. Similarly, this study showed that the TFC contents of 14 species of *Indocalamus* in different seasons ranged from 9.88 mg·g^−1^ to 36.97 mg·g^−1^. Flavonoids guard plant tissues from high-energy wavelengths, thus protecting PSII and chloroplasts of shade plants from high-intensity sunlight [42,43,44]. Angmo [45], Martínez-Lüscher [46], and Szymborska-Sandhu [47] have shown that TFC accumulation decreases when plants are in low-light environments and increases when light intensity increases. However, it has also been shown that there exists an optimum light intensity for TFC accumulation, with *Lithocarpus litseifolius* having the highest TFC contents in leaves at 40% shade [48]. In this study, the annual average TFC contents of 12 species of *Indocalamus* (excluding Lat and Jin) under the ST treatment were reduced compared to those of plants under the CK treatment, probably because these bamboo species do not need to produce more TFC to protect themselves from radiation when the light intensity is low, and 50% full light may be the ideal light intensity to promote the accumulation of TFC in Lat and Jin. The results of the study by Ni [49] showed that the peak contents of active compounds such as TFC in bamboo leaves were generally higher in autumn and winter than in spring and summer and that the best season for harvesting was winter; Ko [50] found a gradual increase in TFC contents in the dwarf bamboo *Sasa quelpaertensis* starting in October and that the TFC contents reached the highest level in December. In this study, under the CK treatment, the 14 species of *Indocalamus* accumulated the most TFC in the winter. Under the ST treatment, the TFC contents of seven species of *Indocalamus* (Dec, Jin, Ped, Fum, Mig, Lat, and Mol) were highest in autumn, and the TFC contents of the remaining species of Indo bamboo peaked in winter, which indicated that the low-light environment affected the complex metabolism of TFC in *Indocalamus*, resulting in differences in TFC accumulation in the leaves of different bamboo species. Phenolics are known to act as protective substances or elicitors that regulate plant–environment interactions, effectively controlling various steps of cell growth and differentiation [51,52]. Zhang [53] has shown that the total polyphenol contents of the 11 species of *Indocalamus* ranged from 0.17% to 0.62%. This study found similar results: the TPC contents of 14 species of *Indocalamus* in different seasons ranged between 1.27 mg·g^−1^ and 5.41 mg·g^−1^. Ntobela [54] demonstrated that low-light environments induced an increase in the contents of volatiles and phenolics, and Formisano [55] found that a low-light environment can promote the accumulation of total phenolic compounds. In this study, after the ST treatment, the annual average TPS contents of eight species of *Indocalamus* increased significantly compared to those under the CK treatment, with Jin, Dec, and Hir showing a lesser degree of decrease and only Kun, Bas, and Hun exhibiting significant decreases. These differences in response may be attributed to the decomposition rate of polyphenolics and leaf cell activity in different bamboo species. The study by Neményi [56] demonstrated that the TPC of four species of Moso bamboo peaked from September to November. In this study, under the CK treatment, the TPC contents of all 14 species of *Indocalamus* were highest in winter, which is consistent with the results of the related studies cited above. Under the ST treatment, the TPC of four species of *Indocalamus* (Fen, Sab, Jin, and Bas) was highest in spring, and the TPC contents of the remaining ten species of *Indocalamus* peaked in winter. The higher contents of active compounds in winter could be attributed to the lower temperature in the winter season, low-temperature stress promoting the accumulation of secondary metabolites, and cold taming leading to an increase in active compounds [57], which was consistent with the general pattern of secondary metabolite transformation and accumulation. The TPC contents of the leaves of some *Indocalamus* species peaked in spring, indicating that the low-light environment can reduce the defense mechanisms of these bamboo species when they are exposed to a cold environment to a certain extent, resulting in a weaker response of their metabolic activities to low-temperature stress [58].

Plant polysaccharides are carbohydrates composed of many different types and several distinct forms of monosaccharides [59,60]. Li [61] has pointed out that the total polysaccharide contents of the three species of *Indocalamus* in winter and summer ranged from 3.59% to 5.95%. This study involved more species and variables, so there are differences in the findings that the TPS contents of 14 species of *Indocalamus* in different seasons ranged between 7.57 mg·g^−1^ and 89.13 mg·g^−1^. Research such as that by Yu [62], Ma [63], and Zheng [64] has demonstrated that the accumulation of polysaccharides is considerably increased in low-light environments. In this study, compared with the CK treatment, the low-light environment increased the average TPS contents and annual average TPS contents of 14 species of *Indocalamus* in summer and winter, probably due to the lower light saturation point of *Indocalamus*. The weaker light intensity is more suitable for the primary metabolism of these species, which is more favorable to the accumulation of carbohydrates. Excluding Fen, Lac, and Hir, the TPS contents of the other 11 species of *Indocalamus* were significantly reduced under the ST treatment in autumn, probably due to the higher precipitation, increase in cloudy and rainy weather, reduction in sunlight hours, and weaker light intensity in autumn; the resulting shading further weakened photosynthesis, inhibiting the synthesis and accumulation of TPS [65]. Under the CK treatment, the TPS of 12 species of *Indocalamus* was highest in autumn, and the TPS contents of Lac and Hir peaked in spring and summer, respectively, which might be caused by the differences in the growth characteristics of these species. After the ST treatment, the time of peak TPS accumulation in the leaves of *Indocalamus* was highly differentiated, with five species of *Indocalamus* (Mol, Jin, Sab, Lat, and Mig) accumulating the most TPS in summer, three species of *Indocalamus* (Dec, Hun, and Lac) accumulating the most TPS in winter, and the remaining six species accumulating the most TPS in autumn. The reason for the peak TPS contents of some bamboo species occurring in summer may be that the ST treatment increased the environmental humidity and soil moisture contents, which reduced the inhibitory effect of high temperature on their photosynthetic assimilation as well as leaf scorching and improved their photosynthetic efficiency, resulting in an increase in synthesized TPS [66]. 

## 5. Conclusions

This study investigated the effects of a low-light environment on the growth adaptability (reproductive ability, morphological characteristics, and photosynthetic properties) and leaf-active substance (TFC, TPC, and TPS) accumulation of 14 species of *Indocalamus*, as well as the seasonal variations in leaf active substance contents in two light environments. The 50% full light treatment enhanced the diameter, height, area per leaf, and LAI of *Indocalamus*, indicating that *Indocalamus* has strong growth adaptability to the low-light environment in the understory space. Different light environments and harvesting times had significant effects on the active substance contents of the leaves of *Indocalamus*. The 50% full light treatment increased the average TFC in autumn, the average TPC in spring, and the average TPS in summer and winter, and the contents of active components in leaves were generally higher in autumn and winter, which was the best season for harvesting leaves. The leaves of Hun and Lac had the highest TFC and TPC contents, while the leaves of Fum had the highest TPS contents. This study provides a theoretical basis for the further development of the leaf resources of *Indocalamus* and their application to sustainable cultivation in the understory, which can help breeders develop unique programs and highlight their potential economic value.

## Data Availability

Data are contained within the article.

## Nomenclature

LAI	leaf area index
P_n_	net photosynthetic rate (μmol CO_2_·m^−2^·s^−1^)
TChl	total chlorophyll (mg·g^−1^)
Chl a/b ratio	chlorophyll a/b ratio
TFC	total flavonoid (mg·g^−1^)
TPC	total polyphenol (mg·g^−1^)
TPS	total polysaccharide (mg·g^−1^)

## Appendix A

**Table A1:** Detailed description of 14 species of *Indocalamus*.

Origin	Bamboo Specie	Abbreviations	Photos	Characterisation
Songming County, Yunnan, China	*I. jinpingensis*	Jin		Culms 1.5–2.0 m tall, 0.4–0.8 cm in diameter; leaf blades oblong-lanceolate, 17–27 cm long, 3.5–5.5 cm wide
	*I. guangdongensis* var. *mollis*	Mol		Culms 1.5–3.0 m tall, 0.9–1.5 cm in diameter; leaf blades broadly lanceolate, 15–35 cm long, 4.0–6.5 cm wide
	*I. kunmingensis* cv. fuminer	Fum		Culms 2.0–3.0 m tall, 0.8–1.8 cm in diameter; leaf blades oblong-lanceolate, 14–40 cm long, 2.2–7.0 cm wide
	*I. migoi*	Mig		Culms 1.5–2.5 m tall, 0.5–1.2 cm in diameter; leaf blades oblong-lanceolate, 20–38 cm long, 3.5–8.0 cm wide
	*I. kunmingensis*	Kun		Culms 1.5–2.5 m tall, 0.5–1.0 cm in diameter; leaf blades oblong-lanceolate, 15–35 cm long, 3.0–6.0 cm wide
	*I. sabaensis*	Sab		Culms 1.0–1.8 m tall, 0.5–1.0 cm in diameter; leaf blades broadly lanceolate, 20–38 cm long, 3.0–7.5 cm wide
	*I. fengsaliensis*	Fen		Culms 1.2–2.0 m tall, 0.7–1.0 cm in diameter; leaf blades broadly lanceolate, 25–38 cm long, 3.5–8.0 cm wide
	*I. pedalis*	Ped		Culms 30–60 cm tall, 0.2–0.4 cm in diameter; leaf blades oblong-lanceolate, 6.5–28 cm long, 1.0–4.0 cm wide
Anji County, Zhejiang Province, China	*I. latifolius*	Lat		Culms 1.2–2.0 m tall, 0.5–1.5 cm in diameter; leaf blades oblong-lanceolate, 10–45 cm long, 2.0–9.0 cm wide
	*I. bashanensis*	Bas		Culms 1.5–3.0 m tall, 1.0–1.5 cm in diameter; leaf blades oblong-lanceolate, 25–36 cm long, 3.0–8.0 cm wide
	*I. hunanensis*	Hun		Culms 1.0–1.8 m tall, 0.4–0.8 cm in diameter; leaf blades linear-lanceolate, 10–28 cm long, 4.5–7.5 cm wide
	*I. decorus*	Dec		Culms 35–80 cm tall, 0.3–0.5 cm in diameter; leaf blades oblong-lanceolate, 15–35 cm long, 3.0–5.5 cm wide
	*I. lacunosus*	Lac		Culms 30–70 cm tall, 0.4–0.6 cm in diameter; leaf blades linear-lanceolate, 10–20 cm long, 3.0–5.0 cm wide
	*I. hirtinodus*	Hir		Culms 30–50 cm tall, 0.2–0.4 cm in diameter; leaf blades oblong-lanceolate, 8.0–15 cm long, 3.0–5.0 cm wide
